# Metabolic engineering of *Escherichia coli* for efficient degradation of 4-fluorophenol

**DOI:** 10.1186/s13568-022-01396-9

**Published:** 2022-05-14

**Authors:** Lijuan Wang, Rihe Peng, Yongsheng Tian, Jing Xu, Bo Wang, Hongjuan Han, Xiaoyan Fu, Jianjie Gao, Quanhong Yao

**Affiliations:** Shanghai Key Laboratory of Agricultural Genetics and Breeding, Biotechnology Research Institute of Shanghai Academy of Agricultural Sciences, 2901 Beidi Road, Shanghai, China

**Keywords:** Multi-monocistronic, 4-Fluorophenol, β-Ketoadipate, Degradation, 4-Substituted phenol

## Abstract

As a kind of refractory organic pollutant, 4-fluorophenol (4-FP) can be degraded by only a few microorganisms with low efficiency because of the great electron-withdrawing ability of fluorine atoms. So it is necessary to artificially construct engineered strain to improve the degradation efficiency and meet the requirements of pollutant degradation. In this study, four genes (*fpdA2*, *fpdB*, *fpdC*, and *fpdD*) for 4-FP degradation from *Arthrobacter sp.* strain IF1 were optimized and synthesized and then reconstructed into *Escherichia coli* by a multi-monocistronic vector to obtain recombinant BL-*fpd* that could degrade 4-FP efficiently. Under optimized induction conditions (inducing the strain by 2 g/L L-arabinose and 1 mM IPTG at 37 ℃), BL-*fpd* could completely degrade 2 mM 4-FP, 4-chlorophenol, 4-bromophenol, and 4-nitrophenol into β-ketoadipate, which could be further metabolized by the bacteria. FpdA2 showed the highest activity towards 4-bromophenol. The strain could completely degrade 1 mM 4-FP in industrial wastewater within 3 h. This study provided a promising strain for the degradation of 4-FP and some other 4-substituted phenols. The construction technologies of multi-monocistronic expression vector may also be used to construct other organic pollutants degrading bacteria.

**Supplementary Information:**

The online version contains supplementary material available at 10.1186/s13568-022-01396-9.

## Introduction

Fluoroaromatic compounds have been increasingly used in the commercial production of pesticides and pharmaceuticals in recent decades (Inoue et al. 2020). These compounds often appear as pollutants in water and soil, causing great harm to the ecological environment and becoming a major environmental problem (Kiel et al. 2015). The introduction of fluorine atoms with strong electron-withdrawing nature on the benzene ring causes considerable changes in the oxidation potential, thus blocking the enzymatic oxidation at the specific location (Park et al. 2001). Therefore, fluorinated aromatic compounds are not easily degraded by microorganisms. So far, only a few microorganisms have been reported to be able to degrade fluoroaromatic compounds, among which *Rhodococcus sp.*, *Pseudoocardia benzenivorans*, *Arthrobacter sp.* strain IF1, *Burkholderia fugorum* FLU100 were involved in the degradation of monofluorophenols (Solyanikova et al. 2003; Ferreira et al. 2008; Kim et al. 2010; Duque et al. 2012; Strunk and Engesser 2013).

At present, two 4-fluorophenol (4-FP) metabolic pathways have been reported: one is the catechol pathway that involves aromatic ring cleavage followed by defluorination. 4-FP is first catalyzed by phenol hydroxylase to produce fluorocatechol. After defluorination by dehalogenase, the aromatic ring is cleaved by catechol 1,2-dioxygenase (Kim et al. 2010). The other is the hydroquinone approach with defluorination followed by aromatic ring cleavage: 4-FP is first dehalogenated by monooxygenase. The hydroquinone produced is further aerobically metabolized by direct ring cleavage, or by hydroxylation to generate hydroxyquinol, which is then cleaved under the catalysis of hydroxyquinol dioxygenase. Then the product β-ketoadipate is metabolized in many steps and eventually enters the tricarboxylic acid cycle (Ferreira et al. 2009; Wells and Ragauskas 2012). No matter which metabolic pathway, the process involves two key steps—aromatic ring cleavage and defluorination.

Biodegradation is considered to be an economical and environmentally friendly way to remove refractory organic compounds from the environment (Peng et al. 2014). At present, although some 4-FP degrading bacteria have been isolated and the biodegradation mechanisms have been revealed, their 4-FP degradation efficiency is usually low, making it difficult to meet the requirements for the treatment of pollutants. The construction of genetically engineered bacteria is an alternative way to improve the efficiency of pollutant degradation. In addition, in the process of bioaugmentation, the highly efficient engineered bacteria have a considerable effect on improving the impact resistance of the pollutant treatment system (Watanabe et al. 2002). With the rapid development of metabolic engineering, a complete heterologous metabolic pathway can be introduced into the host strain rather than a single gene by using synthetic biology technology to design, modify and combine various catalytic elements, thus endowing the engineered bacteria with new degradation functions. This idea has been successfully implemented in practice. Gong Ting et al. (2016) used *Pseudomonas putida* KT2440 as the chassis cell and combined seven genes into the same strain through synthetic biology, to create a multi-functional degradation strain capable of degrading methyl parathion, *γ*-hexachlorocyclohexane and their intermediate products at the same time.

**Fig. 1:**
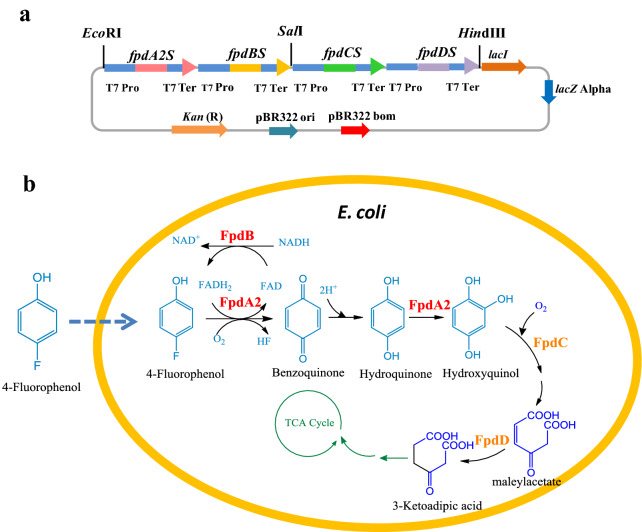
4-FP degradation pathway constructed in *E. coli.* **a** Schematic representation of the multi-monocistronic vector pC1301-*fpdA2BCDS*; **b** Metabolism of 4-FP in recombinant strain BL-*fpd*. FpdA2, 4-FP monooxygenase; FpdB, flavin reductase; FpdC, hydroxyquinol dioxygenase; FpdD, maleylacetate reductase

In this study, the entire 4-FP degradation system of *Arthrobacter sp.* strain IF1 including the defluorination module and the aromatic ring cracking module was optimized and reconstructed into *E. coli*, endowing it with the functions of degrading and tolerance to 4-FP (Fig. 1b). In addition, the factors affecting the protein expression activity and degradation efficiency of the engineered bacteria were optimized, and the degradation substrate spectrum was analyzed.

## Material and methods

### Reagents

β-Ketoadipate was obtained from Finetech Industry Limited (Wuhan, China). Molecular biology experimental reagents were purchased from TaKaRa Biotechnology (Dalian, China) Co. unless otherwise noted. Other chemicals used in the study were purchased from J&K Scientific Ltd., China.

### Construction of 4-FP degradation strain BL*-fpd*

The genes used for 4-FP degradation in this study included *fpdA2*, *fpdB*, *fpdC* (Genbank: AB530681.1), and *fpdD* (Genbank: AB530680.1) from *Arthrobacter sp.* strain IF1. The above four gene sequences were optimized and analyzed by online tools (GenSmart™ Codon Optimization, https://www.genscript.com/tools/gensmart-codon-optimization; http://www.detaibio.com/tools/index.php?r=site%2Findex), and then checked and modified manually. These genes were optimized with *E. coli* preference codon. The specific recognition sites of endonucleases in the gene sequences were eliminated to facilitate vector construction. GC content was balanced. Reverse repeat sequences of genes or adjacent genes within 200 bp and stem-loop structures were removed to improve the mRNA stability. The optimized genes named as *fpdA2S* (Genbank: OM108470), *fpdBS* (Genbank: OM108471), *fpdCS* (Genbank: OM108472), *and fpdDS* (Genbank: OM108473) were chemical synthesized and verified by DNA sequencing (Sangon Biotech Co., China).

Two gene expression cassettes named T7*fpdA2S*–T7*fpdBS* and T7*fpdCS*–T7*fpdDS* were constructed by connecting T7 promoter (5’-CTCGAGCGATCCCGCGAAATTAATACGACTCACTATAGGGGAATTGTGAGCGGATAACAATTCCCCTCTAGAAATAATTTTGTTTAACTTTAAGAAGGAGATATACC) at the 5'-end and T7 terminator (5’-CTAGCATAACCCCTTGGGGCCTCTAAACGGGTCTTGAGGGGTTTTTTGGTCGACGGTGACGTTGAGCATGGT) at the 3'-end of each gene using an improved overlapping extension PCR method (Peng et al. 2006). The primers for gene expression cassettes construction were shown in Additional file 1: Table S1 and the PCR conditions were shown in Additional file 1: Fig. S1. A modified vector pCAMBIA1301 harboring the lactose operon repressor (*lacI*) gene (Genbank: MK720606.1) (Wang et al. 2019) was used to construct the expression vector, so that IPTG could be used to enhance the regulation of protein expression. This vector was suitable for the expression of long fragment genes. Two cassettes T7*fpdA2S*–T7*fpdBS* and T7*fpdCS*–T7*fpdDS* were successively inserted into the *Eco*RI/*Sal*I sites and *Sal*I/*Hin*dIII sites of the modified vector pCAMBIA1301 by twice ligations and transformations. The correct construction of the expression vector was identified by restriction enzyme digestion and sequencing. The obtained multi-monocistronic expression vector named pC1301-*fpdA2BCDS* was then transformed into *E. coli* BL21-AI (Invitrogen, USA) to construct the strain BL-*fpd* (Fig. 1a). *E. coli* BL21-AI carrying the modified pCAMBIA1301was named as BL-*control*.

### Culture conditions

*E. coli* BL-*fpd* was inoculated into 50 mL of LB medium (10 g tryptone, 5 g yeast extract, and 10 g NaCl per liter) containing 50 mg/L kanamycin and cultured at 37 ℃ until *OD*_600_ reached 0.6. Then the cells were collected washed with double distilled water and resuspended with the same volume of M9 medium (10 g glycerol, 6 g Na_2_HPO_4_, 3 g KH_2_PO_4_, 1 g NH_4_Cl, 0.5 g NaCl, 0.5 mmol MgSO_4_, 0.1 mmol CaCl_2_, and 5 g acid-hydrolyzed casein per liter) containing 50 mg/L kanamycin. The inducer containing L-arabinose and isopropyl-β-D-thiogalactoside (IPTG) was added (or not added) to the culture, and the bacteria were induced at different temperatures. BL-*control* was used as control.

### Gene expression analysis

BL-*control* and BL-*fpd* were cultured with LB medium until *OD*_600_ reached 0.6 at 37 ℃, and their plasmids were extracted. The specific fragments of the four genes were amplified by PCR using the extracted plasmids as templates and identified by DNA sequencing. Total RNA from *E. coli* BL-*fpd* and BL-*control* were extracted after 3 h of induction using the RNA extraction kit (TRIzol) according to the manufacturer's manual. The cDNA was synthesized at 42 ℃ for 15 min by TransScript® One-Step gDNA Removal and cDNA Synthesis SuperMix. The Quantitative real-time polymerase chain reaction (qRT-PCR) was performed on the Bio-Rad MJ Mini personal thermal cycler using SYBR Premix Ex Taq II (Takara Bio Inc.). The *E. coli 16S* rRNA gene (Genbank: NR_024570.1) was used as an internal control. The specific primers used for PCR/qRT-PCR and PCR/qRT-PCR conditions were shown in Additional file 1: Table S2. The relative expression values of the genes were calculated by 2^–ΔCT^ = 2^−[CT(target)−CT(16S)]^ (Wang et al. 2019).

### Study on protein expression and biodegradation conditions

The effect of induction temperature on the degradation capacity of 4-FP of the strain BL-*fpd* was investigated by adding 2 mM 4-FP and 10 μM FAD^+^ to the culture after 3 h of induction at different temperatures (25 ℃, 30 ℃ and 37 ℃). The effect of inducer concentration (low dose inducer 1: 0.2 g/L final concentration of L-arabinose and 0.1 mM final concentration of IPTG; high dose inducer 2: 2 g/L final concentration of L-arabinose and 1 mM final concentration of IPTG) on 4-FP degradation was also investigated. The degradation of 4-FP over time and intermediate metabolites (hydroquinone, hydroxyquinol), the product inorganic fluoride and β-ketoadipate were detected. The concentration of inorganic fluorine in the supernatants was measured by an ion meter (PXSJ-227L, INESA Scientific Instrument Co., Ltd., China) equipped with a fluoride electrode (PF-202-L, INESA). Sodium fluoride standard was prepared for the calibration curve. Analysis for the concentrations of 4-FP, hydroquinone and hydroxyquinol were determined by high performance liquid chromatography (HPLC) as described below, and the concentrations of β-ketoadipate were detected by gas chromatography-mass spectrometry (GC–MS).

### Assay of 4-FP tolerance

*E. coli* BL-*fpd* was induced for 3 h with inducer 2 in M9 medium at 37 ℃, followed by adding 4-FP of different concentrations (0, 2, 4, 6, 8 mM) and 10 μM FAD^+^. Cell growth was measured by the optical density of the culture at 600 nm.

The cell morphology of the strains was examined by scanning electron microscope (SEM) (TM4000 plus, Hitachi). BL-*control* and BL-*fpd* were induced for 3 h and then treated with 4 mM 4-FP for 12 h. The cells were collected and pre-treated according to the method in reference (Rocha et al. 2011).

### Degradation substrate spectrum of BL-*fpd*

To study the degradation substrate spectrum of strain BL-*fpd*, a series of organic compounds (4-FP, 4-chlorophenol, 4-bromophenol, 4-nitrophenol, and hydroquinone) were tested as substrates. BL-*fpd* was induced for 3 h with inducer 2 at 37 ℃, followed by adding 2 mM of various substrates and 10 μM FAD^+^. The degradation of each substrate was analyzed by HPLC after 3 h. The strain BL-*control* was used as a control.

### Biodegradation of 4-FP in wastewater

To test the biodegradation ability of BL-*fpd* to 4-FP in wastewater, the wastewater mainly containing 4-FP from a chemical plant in Changzhou, China, was used for the degradation by engineered bacteria. The content of 4-FP in the wastewater was 0.12 mM and the pH value was 6.5. The reaction mixture consisted of 4 mL wastewater containing 4-FP (the concentration of 4-FP was artificially increased to 1 mM), 10 μM FAD^+^ and 1 mL bacterial culture after 8 h induction by inducer 2. The content of residual 4-FP was determined by HPLC after 3 h reaction at 37 °C.

### HPLC and GC–MS analysis

Compounds were analyzed by HPLC equipped with an ultraviolet spectrophotometric detector (Agilent 1100 VWD) and an Athena C18 reversed-phase column (250 mm × 4.6 mm × 5 μm, ANPEL Inc., China) at 30 ℃. To detect 4-FP, 20 μL samples were eluted at a flow rate of 1 mL/min with a solution of water/acetonitrile (70:30) and monitored at 223 nm. Hydroquinone and hydroxyquinol were analyzed at 280 nm using a 5 − 30% linear gradient of acetonitrile for 20 min, and the flow rate was 0.5 mL/min. Calibration curves were plotted by peak area versus concentration of each standard. The detection conditions of 4-chlorophenol and 4-bromophenol were as follows: The mobile phase was water/methanol (30:70) at a flow rate of 0.8 mL/min, and the detection wavelength was 280 nm. 4-nitrophenol was detected at 280 nm using a mobile phase of water/methanol (45:55) at a flow rate of 0.5 mL/min.

β-Ketoadipate in the culture was treated and detected by GC–MS regarding the method used by Okamura-Abe et al. (2016). Briefly, 500 μL culture was acidified to below pH 2 with concentrated hydrochloric acid and extracted twice with 500 μL ethyl acetate (benzoic acid as internal standard). Then 200 μL of the organic phase was evaporated for further steps. The samples were derivatized by trifluoroacetamide and determined by GC–MS/MS 7890B-7000C system (Agilent) equipped with an HP-5 MS column (30 m × 0.25 mm × 0.25 μm, Agilent). The column oven temperature increased from 100 ℃ to 160 ℃ with a rate of 40 ℃/min, then 10 ℃/min from 160 ℃ to 250 ℃ and 20 ℃/min from 250 ℃ to 300 ℃. For β-ketoadipate identification, the derived sample mass spectra were compared with the corresponding standard spectra. The standard curve of peak area versus concentration was used to determine the concentration of β-ketoadipate.

## Results

### Construction of 4-FP degradation pathway in *E. coli*

To improve the transcription efficiency of genes in *E. coli*, codon optimization was performed on four key genes in the 4-FP degradation pathway and renamed as *fpdA2S*, *fpdBS*, *fpdCS*, and *fpdDS*. With the same amino acid sequences, the identities of the optimized four nucleotide sequences compared with the original sequences were 80.66%, 77.96%, 78.41%, and 78.83%, respectively. Among them, *fpdA2S* and *fpdBS* genes constituted the defluorination module, while *fpdCS* and *fpdDS* genes constituted the aromatic ring cracking module.

**Fig. 2:**
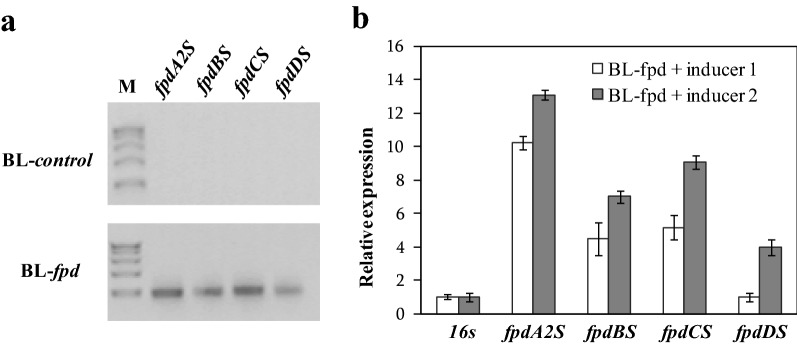
Expression of 4-FP degradation pathway genes. **a** PCR amplified fragments using plasmid from BL-*control* or BL-*fpd* as the template (M, DL2000). The plasmids were extracted when *OD*_600_ reached 0.6 after being cultured in LB medium; **b** Relative transcript level analysis of the exogenous genes in BL-*fpd* at different concentrations of inducers by qRT-PCR. The relative expression values of the genes (relative to the internal control *16S* gene) were calculated by 2^–ΔCT^ = 2^−[CT(target)−CT(16S)]^. inducer 1 (0.2 g/L final concentration of L-arabinose and 0.1 mM final concentration of IPTG), inducer 2 (2 g/L final concentration of L-arabinose and 1 mM final concentration of IPTG). Values are the mean ± SD of three replicates

The successful transfer of the four genes into the host was verified by PCR amplification using plasmid from BL-*control* or BL-*fpd* as the template and DNA sequencing. And qRT-PCR was used to analyze the amount of mRNA. As shown in Fig. 2a, the amplified fragments of all genes could be detected in BL-*fpd*. However, the relative mRNA expression values of each gene were not consistent (Fig. 2b). The concentration of inducer also affected the mRNA expression. Compared with low dose inducer 1, the mRNA expression values of all genes increased under high dose inducer 2. Most notably, the mRNA level of *fpdDS* increased about 3 times. The mRNA expression of *fpdA2S* was the highest under inducer 1, which was 10.26 times that of *fpdDS* with the lowest expression. Under the induction of inducer 2, the mRNA expression difference between the two genes was reduced to 3.29 times.

### Biodegradation of 4-FP by BL*-fpd* in vivo

**Fig. 3:**
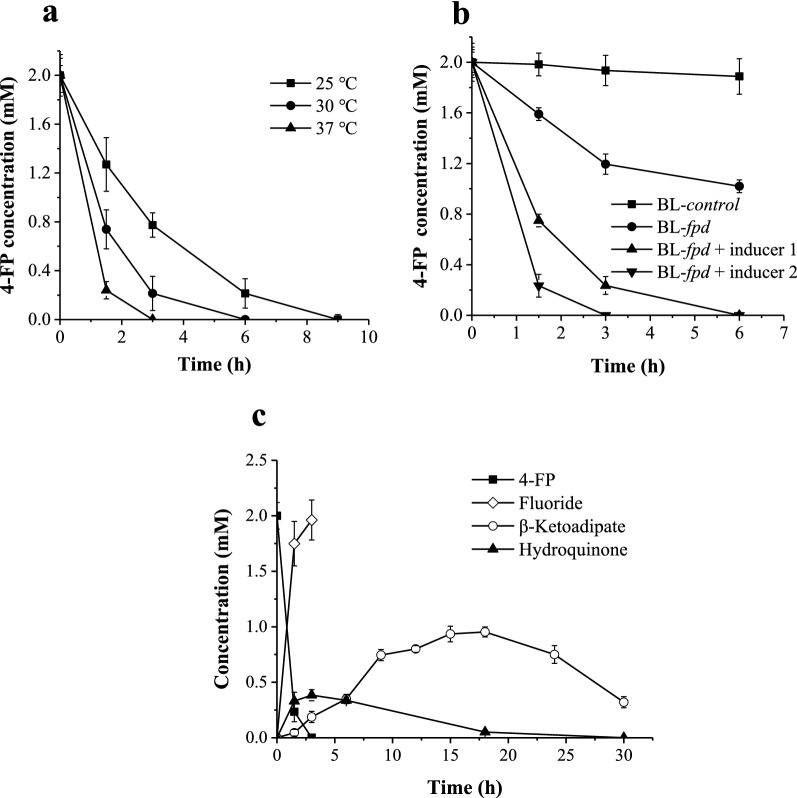
Degradation of 4-FP by BL-*fpd*. **a** Optimization of induction temperature under inducer 2; **b** Optimization of inducer concentration at 37 ℃; **c** Time-course curve of 4-FP degradation metabolites by BL-*fpd* under optimized conditions (37 ℃, inducer 2)*.* The data are the mean ± SD for three independent experiments

The induction conditions of BL-*fpd* for intracellular degradation of 4-FP were optimized. As shown in Fig. 3a, it took only 3 h for 2 mM 4-FP to be completely degraded by BL-*fpd* at 37 °C induction temperature under inducer 2. While at 25 °C and 30 °C, the degradation time was extended to 9 h and 6 h, respectively. Figure 3b showed that the addition of high dose inducer 2 was more beneficial to the expression of the degrading enzyme system, thus promoting the degradation of 4-FP. 4-FP could be degraded to a certain extent in the absence of the inducer, which may be due to the unavoidable leakage of protein expression in the T7 expression system (Kato 2020).

As shown in Fig. 3c, 98% of the inorganic fluoride could be detected with the conversion of 2 mM 4-FP in 3 h, indicating that 4-FP was degraded efficiently. The accumulation of intermediate metabolite hydroquinone indicated its degradation as a rate-limiting step throughout the process. Under optimized induction conditions, the hydroquinone content accumulated rapidly within 4 h, then decreased and completely degraded within 30 h. In addition, the intermediate product hydroxyquinol was not detected at all sampling points, indicating that hydroxyquinol generated in the previous reaction was rapidly transformed in BL-*fpd*. β-Ketoadipate, the final product of 4-FP degradation, was detected by GC–MS (Additional file 1: Fig. S1). As shown in Fig. 3c, the concentration of β-ketoadipate in BL-*fpd* culture reached the maximum value of 0.96 mM after adding 2 mM 4-FP for 18 h. After that, its concentration decreased gradually with the metabolism and utilization of cells.

The coenzyme regeneration system of the cell itself, such as NADH, could effectively reduce the cost of degradation reaction. However, FAD^+^ in the cell was insufficient to support the rapid degradation of 4-FP, and exogenous FAD^+^ must be added to ensure the reaction according to our experiments.

### 4-FP tolerance of *E. coli *BL*-fpd*

**Fig. 4:**
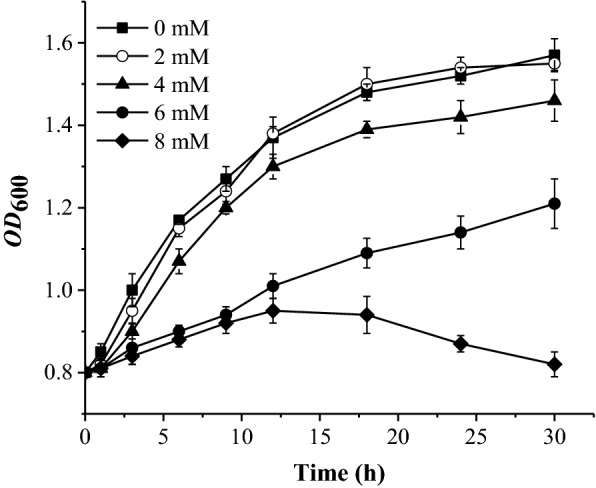
Growth curves of BL-*fpd* with different concentrations of 4-FP. The data are the mean ± SD for three independent experiments

The *OD*_600_ value was used to reflect the tolerance of cells to various concentrations of 4-FP. *E. coli* showed strong tolerance to 4-FP by introducing the 4-FP metabolic pathway (Fig. 4). After culturing for 30 h with 4 mM of 4-FP, the *OD*_600_ value still reached 93% of that without 4-FP, while the growth of BL-*fpd* was seriously inhibited by adding 8 mM of 4-FP. The inhibition effect of 4-FP on BL-*fpd* was enhanced with the increase of 4-FP concentration.

**Fig. 5:**
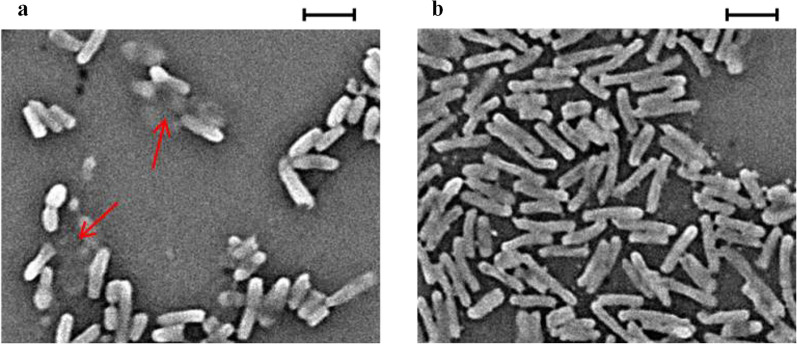
Scanning electron micrographs of BL-*control* and BL-*fpd* after 4-FP treatment. **a** View of BL-*control* treated with 4 mM 4-FP for 12 h (average cell length: 1.639 μm). The location of viscous substances attached to the cell surface was marked by red arrow; **b** View of BL-*fpd* treated with 4 mM 4-FP for 12 h (average cell length: 1.921 μm, P < 0.05); SEM operating parameters: 15 kV 7.2 mm × 2.50 K. Scale bars: a–b 2 μm

The growth morphology of BL-*control* and BL-*fpd* was observed by SEM to further evaluate the damage of the cells by 4-FP treatment. After 12 h of treatment with 4 mM 4-FP, the BL-*control* cells became shorter and coarser with an average length of 1.639 μm, accompanied by viscous substances attached to the cell surface (Fig. 5a). While most of the BL-*fpd* cells showed normal morphology, and the average length was 1.921 μm (Fig. 5b). The introduction of exogenous 4-FP degrading genes enhanced the tolerance of *E. coli* to 4-FP, which was beneficial to the efficient remediation of 4-FP pollutants.

### Degradation substrate spectrum of BL-*fpd*

**Fig. 6:**
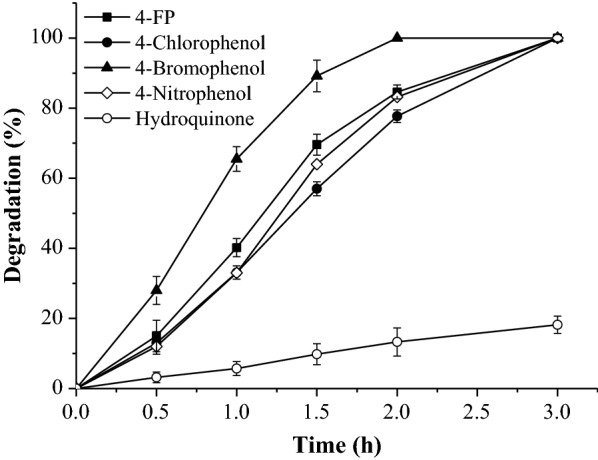
Degradation of some 4-substituted phenols and hydroquinone by BL-*fpd*. The data are the mean ± SD for three independent experiments

The degradation of some 4-substituted phenols and hydroquinone was investigated. As shown in Fig. 6, the strain BL-*fpd* also showed strong degradation capacity for some other 4-substituted phenols in addition to 4-FP. 4-Chlorophenol, 4-bromophenol, and 4-nitrophenol could be completely degraded in 3 h, and FpdA2 showed the highest activity towards 4-bromophenol among the substrates tested. The metabolite of these 4-substituted phenols after the first step degradation, as well as that of 4-FP, was hydroquinone. Then hydroquinone was further degraded into β-ketoadipate which could be metabolized by the strain (Fig. 3c). The degradation of hydroquinone was only 18.2%, and hydroquinone hydroxylation was once again verified as a rate-limiting step in the degradation pathway.

### Biodegradation of 4-FP in wastewater

To verify the actual degradation efficiency of recombinant *E. coli* in wastewater containing 4-FP, the wastewater containing 1 mM 4-FP was added to BL-*fpd* culture in a 4:1 ratio and incubated at 37 °C. 1 mM 4-FP in wastewater could be completely degraded in 3 h (Additional file 1: Fig. S3). In this process, a decrease in pH value of the reaction system was detected, which was due to the degradation of 4-FP accompanied by the release of HF and its complete dissolution in water (Ferreira et al. 2008; Yang et al. 2013). In industry, coagulation/precipitation methods, adsorption processes and ion exchange processes can be used to remove fluoride from wastewater (Solanki et al. 2022; Yadav et al. 2017).

## Discussion

Fluorinated compounds are rare in nature. In the decades since the fluoroaromatic compounds have been created by humans, only a few microorganisms can evolve to degrade them in the natural environment because of the great electron-withdrawing ability of fluorine atoms (Park et al. 2001). So it is particularly important to artificially construct engineered strains containing different degradation modules of fluoroaromatic compounds to meet the requirements of pollutant degradation. Due to the clear genetic background and mature protein expression system, *E. coli* was often used as a bacterial chassis for biodegradation studies (Tran et al. 2021). For example, Yang et al. (2016) introduced the *mdeABCD* gene cluster involved in methyl oxidation from *Hydrogenophaga atypical* QY7-2 into *E. coli* BL21 (DE3), and successfully constructed an engineered strain for degrading 3-methyl diphenyl ether. However, even in the model strain of *E. coli*, the co-expression of multiple genes presents many challenges such as the control of the consistency of the multi-gene expression level and the stability of the transferred plasmid (Perrakis and Romier 2008). In particular, the four genes involved 4-FP degradation were derived from two gene clusters with lengths of 5,145 bp and 9373 bp in *Arthrobacter sp.* strain IF1, respectively. Therefore, it is difficult to construct the degradation pathway by directly cloning gene clusters.

To successfully clone and express multiple genes in the degradation pathway, the construction technologies of multi-monocistronic expression vector were applied in this study. First, we optimized the codons and mRNA structure of multi-gene to improve the stability and efficiency of gene transcription and translation. Secondly, by connecting independent T7 promoter and terminator at both ends of each gene fragment, two gene expression cassettes containing multi-gene were constructed to eliminate the weakening of expression levels caused by the long distance between promoters and gene sequences (Shrestha et al. 2019). Thirdly, the improved overlapping extension PCR technology was used to realize the flexible splicing of gene expression elements instead of enzyme ligation. These techniques for constructing multiple genes into the same vector have certain universality, and can be applied to other organic pollutant degradation systems or microbial modifications as required. At the same time, the whole degradation pathway can also be applied to other safer and more adaptable hosts to construct environmental engineering bacteria.

The engineered strain containing a multi-monocistronic vector has many advantages. This kind of single plasmid system has higher genetic stability than the multi-plasmid expressing multi-gene system and does not need to withstand the pressure of various antibiotics on the strain. While compared with the synergistic degradation of multiple strains, there is no need to consider the transport of intermediate products, and cofactors can be recycled in the cell for maximum utilization. In our laboratory, an engineered strain for phenol degradation has been successfully constructed using this design (Wang et al. 2019). And a similar multi-monocistronic vector system has been used for the synthesis of cyanidin 3‑O‑glucoside (Shrestha et al. 2019).

Compared with the original strain *Arthrobacte sp.* strain IF1 which took 120 h to complete the conversion of 1 mM 4-FP (Ferreira et al. 2008), the engineered strain BL-*fpd* could completely degrade 2 mM 4-FP in 3 h, showing obviously improved degradation efficiency for 4-FP. Due to the non-specific degradation of FpdA2 to 4-substituted phenols, BL-*fpd* also degraded 4-chlorophenol, 4-bromophenol, and 4-nitrophenol into β-ketoadipate. A similar enzyme was NpdA2 from *Arthrobacter sp.* strain JS443, which could degrade 4-nitrophenol and 4-chlorophenol with the release of 4-substituted groups (Perry and Zylstra 2007). The non-specificity of these two enzymes to substrates was also reflected in the conversion of hydroquinone to hydroxyquinol by a second hydroxylation step (Ferreira et al. 2008).

Due to the complexity of components in industrial wastewater, it is difficult for microorganisms that can only degrade single organic pollutants to give full play to their degradation efficiency. The high toxicity of 4-substituted phenols on the microorganisms and their cross inhibition effect tend to limit the bioremediation of environmental pollutants (Saéz and Rittmann 1991; Yuan and Lu 2005). As common organic pollutants in water bodies (Liu et al. 2009), these 4-substituted phenols were non-specifically degraded by BL-*fpd*, which was beneficial for combined remediation of pollutants and improved its application value in bioremediation. In addition, by accurately designing genes, modifying metabolic pathways and combining different metabolic modules using the construction techniques of the multi-gene expression vector in this study, the engineered bacteria can obtain new biodegradation functions. For example, the combination of gene *mpd* from *Stenotrophomonas* (Yang et al. 2006) with four genes in the 4-FP degradation pathway in this study may effectively degrade pesticide Methyl Parathion. Hydroquinone and hydroxyquinol are intermediate metabolites of many phenolic pollutants (Perry and Zylstra 2007; Wells and Ragauskas 2012; Min et al. 2018). Therefore, the key genes of different phenolic metabolites can be combined with the aromatic ring cracking module in this study to obtain super engineered bacteria that can degrade a variety of pollutants.

It is undeniable that there are still some defects in the application of engineering strains in the actual remediation of pollutants, including the lack of adaptability to the complex polluted environment such as high salt, strong acid or strong alkali, and the risk of artificially modified gene fragments escaping into the natural environment (Moe-Behrens et al. 2013). Therefore, the research progress of using genetically engineered bacteria for environmental remediation has been relatively slow in the past ten years. In view of this, the immobilization and biofilm preparation of BL-*fpd* can be implemented to reduce the toxicity of complex environments to cells in a subsequent study (Wang et al. 2002; Patel et al. 2010). In addition, a conditional suicide system may also be established in BL-*fpd* in the future to control its escape into the natural environment (Li et al. 2020).

In this research, the 4-FP degradation pathway was introduced into *E. coli* by a multi-monocistronic expression vector, and an engineered strain for 4-FP degradation was constructed for the first time. The strain could efficiently degrade 4-FP and some other 4-substituted phenol into available carbon sources. This study provided a promising strain for the degradation of 4-FP and some other 4-substituted phenols. The construction technologies of multi-monocistronic expression vector may also be used to construct other organic pollutants degrading bacteria.

## Supplementary Information

**Additional file 1: Table S1**. Primers for gene expression cassettes construction. **Table S2**. Primers for PCR/qRT-PCR in this study. **Fig. S1**. Construction of the gene expression cassettes. **Fig. S2**. GC-MS analysis of β-ketoadipate from 4-FP degradation by BL-fpd. **Fig. S3**. Degradation of 4-fluorphenol in industrial wastewater

## Data Availability

The corresponding author is responsible for providing all experimental data on reasonable request.
