# Supplementary material for: Metabolic engineering of Escherichia coli for efficient degradation of 4-fluorophenol
Source: AMB Express. 2022 May 14;12:55. doi: 10.1186/s13568-022-01396-9 (PMC9107566; doi:10.1186/s13568-022-01396-9)
Supplement: Supplementary file 1 — Additional file 1: Table S1. Primers for gene expression cassettes construction. Table S2. Primers for PCR/qRT-PCR in this study. Fig. S1. Construction of the gene expression cassettes. Fig. S2. GC-MS analysis of β-ketoadipate from 4-FP degradation by BL-fpd. Fig. S3. Degradation of 4-fluorphenol in industrial wastewater [file 13568_2022_1396_MOESM1_ESM.docx]

**Supplementary materials**

**Table S1** Primers for gene expression cassettes construction

|  | **Sequences of the primers** |
| --- | --- |
| P1 | 5’-GAATTCCTCGAGCGATCCCG |
| P2 | 5’-ACTCTTTACCAGTACGCATGGTATATCTCCTTCT |
| P3 | 5’-AGAAGGAGATATACCATGCGTACTGGTAAAGAGT |
| P4 | 5’-CCAAGGGGTTATGCTAGTTATGCAGGTGCAGTCAC |
| P5 | 5’-GTGACTGCACCTGCATAACTAGCATAACCCCTTGG |
| P6 (P18) | 5’-GCGGGATCGCTCGAGACCATGCTCAACGTCACC |
| P7 (P19) | 5’-GGTGACGTTGAGCATGGTCTCGAGCGATCCCGC |
| P8 | 5’-AGTATCAGAGATTGACATGGTATATCTCCTTCT |
| P9 | 5’-AGAAGGAGATATACCATGTCAATCTCTGATACT |
| P10 | 5’-CAAGGGGTTATGCTAGTTAACGACGACCAGGTTCG |
| P11 | 5’-CGAACCTGGTCGTCGTTAACTAGCATAACCCCTTG |
| P12 | 5’-GTCGACACCATGCTCAACGTC |
| P13 | 5’-GTCGACCTCGAGCGATCCCG |
| P14 | 5’-CAGCAGGACGAGTTGACATGGTATATCTCCTTCT |
| P15 | 5’-AGAAGGAGATATACCATGTCAACTCGTCCTGCTG |
| P16 | 5’-CCAAGGGGTTATGCTAGTTACTTCAGATCCAGAGT |
| P17 | 5’-ACTCTGGATCTGAAGTAACTAGCATAACCCCTTGG |
| P20 | 5’-ACTCAAGAAGATCAGTCATGGTATATCTCCTTCT |
| P21 | 5’-AGAAGGAGATATACCATGACTGATCTTCTTGAGT |
| P22 | 5’-CAAGGGGTTATGCTAGTTAGAACTCAGTACCATTC |
| P23 | 5’-GAATGGTACTGAGTTCTAACTAGCATAACCCCTTG |
| P24 | 5’-AAGCTTACCATGCTCAACGTC |

P1-P12 were used for the construction of cassette T7*fpdA2S*–T7*fpdBS.* P13-P24 were used for the construction of cassette T7*fpdCS*–T7*fpdDS.*

**Table S2** Primers for PCR/qRT-PCR in this study

| **Gene** | **Sequences of the primers** |
| --- | --- |
| *fpdA2S* | *fpdA2S-*F 5'-AGGTGGATGGTCCACTGACTC |
|  | *fpdA2S-*R 5'-TGCAGGTGCAGTCACAGCAGA |
| *fpdBS* | *fpdBS -*F 5'-CTGCAAACATGGCACTTGAAC |
|  | *fpdBS* -R 5'-AACGACGACCAGGTTCGTTAC |
| *fpdCS* | *fpdCS* -F 5'-CTGCTGCTGATCATCGTACTC |
|  | *fpdCS*-R 5'-GCACGATGTCGAAACGAGTAC |
| *fpdDS* | *fpdD* -F 5'-ATCTCTAACGCACTTGGTGCA |
|  | *fpdD* -R 5'-ATGAAGCAGGATCAGCAGGTC |
| *16S* | *16S*-F 5'-ACTCCTACGGGAGGCAGCAG |
|  | 16S-R 5'-ATTACCGCGGCTGCTGG |

The PCR was performed with 1 U *TaKaRa* *Taq*, 5 nmol dNTPs, 10 pmol of each primer, and 10 ng plasmid DNA as the template in a final volume of 25 μL. The PCR was carried out at 94°C for 10 min, followed by 20 s at 94 ºC, 20 s at 60 ºC, 20 s at 72 ºC for 30 cycles, and a final extension at 72 ºC for 5min. The qRT-PCR was performed with 12.5 μL SYBR Premix Ex Taq II, 10 pmol of each primer, and 100 ng cDNA as the template in a final volume of 25 μL. The qRT-PCR condition was as follows: 10 min at 94 ºC, followed by 40 cycles of 20 s at 94 ºC, 20 s at 60 ºC, 20 s at 72 ºC, and a final extension at 72 ºC for 5min.


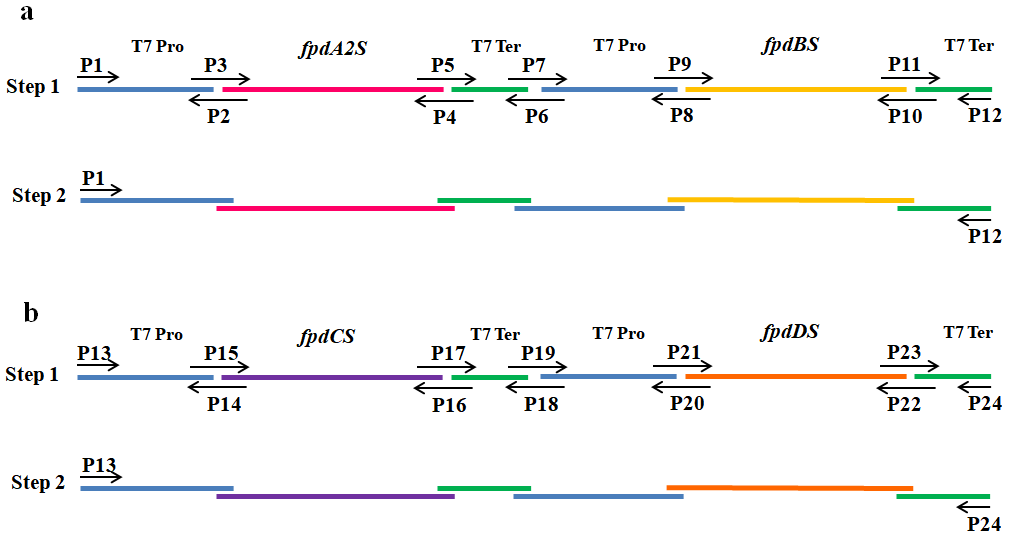


**Fig. S1** Construction of the gene expression cassettes. **a** The cassette T7*fpdA2S*–T7*fpdBS* was constructed using an improved overlapping extension PCR consisted of two-step. Step 1: Six fragments were obtained synchronously by PCR using plasmids containing T7 promotor, *fpdA2S*, T7 terminator and *fpdBS* as templates and six pairs of primers (P1 and P2, P3 and P4, P5 and P6, P7 and P8, P9 and P10, P11 and P12). The first PCRs were performed with 1 U *Pyrobest* DNA polymerase, 10 nmol dNTPs, 30 pmol of each primer, and 10 ng of each plasmid DNA in a final volume of 50 μL. The PCR conditions were 30 s at 94 °C and 60 s at 68 °C for 10 cycles. Step 2: The six DNA fragments obtained by the first-step PCRs were mixed as the template, and P1 and P12 were used as primers. The second PCR was performed with 1 U *Pyrobest* DNA polymerase, 10 nmol dNTPs, 30 pmol of each primer, and 2 pmol template in a final volume of 50 μL. The PCR was carried out at 94°C for 1 min, then 25 cycles of denaturing at 94 °C for 30 s, annealing at 54 °C for 30 s, and extension at 72 °C for 3 min. **b** The cassette T7*fpdCS*–T7*fpdDS* was constructed in the same way.


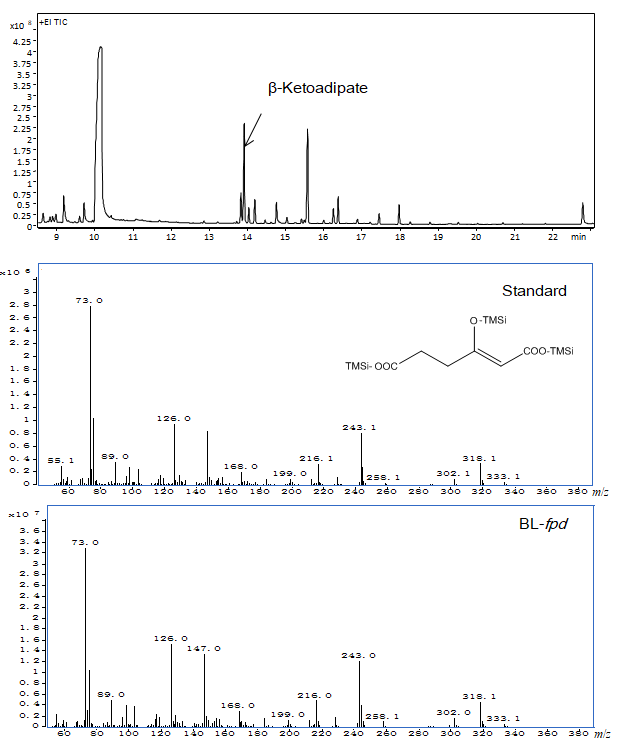


**Fig. S2** GC-MS analysis of β-ketoadipate from 4-FP degradation by BL-*fpd*

**Fig. S3** Degradation of 4-fluorphenol in industrial wastewater*.* The samples were measured by HPLC at 3 h. Peak corresponding to 4-FP appear at R_t_ 6.65 min. **a** Chromatograms of 4-FP standard; **b** Chromatograms of wasterwater containing 1 mM 4-FP; **c** Chromatograms of wastewater treated by BL-*control*; **d** Chromatograms of wastewater treated by BL-*fpd*.
